# Sex- and Dose-Dependent Differences in the Development of an Addiction-Like Phenotype Following Extended-Access Fentanyl Self-Administration

**DOI:** 10.3389/fphar.2022.841873

**Published:** 2022-03-17

**Authors:** Eleanor Blair Towers, Ben Setaro, Wendy J. Lynch

**Affiliations:** Psychiatry and Neurobehavioral Sciences, University of Virginia, Charlottesville, VA, United States

**Keywords:** sex differences, addiction-like phenotype, fentanyl intake and frequency of use, extended intermittent access, self-administration, vulnerability to relapse, physical dependence, opioid use disorder and biological sex

## Abstract

Opioid use disorder (OUD) is a major epidemic in the United States, and fentanyl is a major culprit. The National Institute on Drug Abuse has highlighted an urgent need for research on the risks and outcomes of OUD with fentanyl; a better understanding of sex/gender differences is also critically needed given that the opioid epidemic has been particularly impactful on women. In response to this need, we developed a rat model of OUD with fentanyl and showed that sex impacts relapse vulnerability following extended-access self-administration under a low fentanyl dose. Here, our goal was to determine sex differences across a broad dose range, including high doses expected to maximize the expression of addiction-like features (e.g., vulnerability to relapse and physical dependence). Male and female rats were assigned to self-administer one of four fentanyl doses (0.25, 0.75, 1.5, and 3.0 µg/kg/infusion), and once they acquired, they were given extended (24-h/day), intermittent access (2, 5 min trials/h, fixed-ratio 1) to fentanyl for 10 days. Physical dependence (spontaneous weight loss) was assessed during early withdrawal, and relapse vulnerability was assessed on withdrawal day 15 using an extinction/cue-induced reinstatement procedure. Despite markedly higher intake in the high- versus low-dose groups, each group responded similarly during relapse testing (extinction and cue-induced reinstatement). However, number of infusions, or frequency of use, during extended access was predictive of later vulnerability to relapse, whereas total intake impacted physical dependence given that weight loss only occurred following the discontinuation of fentanyl self-administration at the three highest doses. Females self-administered more fentanyl each day and within each binge (active trial), and had longer lasting weight loss during withdrawal than males. Relapse vulnerability was also higher in females than males and highest in females tested during estrus. These findings indicate that sex is an important risk factor for patterns and levels of fentanyl intake, relapse, and physical dependence, and while fentanyl intake predicts physical dependence, frequency of use predicts relapse.

## Introduction

Opioid use disorder (OUD) is a major epidemic in the United States. The epidemic is intensifying with opioid-involved overdose deaths reaching the highest number ever recorded in the 12-month period leading up to April 2021, which was primarily driven by fentanyl, a synthetic opioid (Centers for Disease Control and Prevention, 2021). The National Institute on Drug Abuse (NIDA) has highlighted an urgent need for research on the risks and outcomes of OUD specifically with fentanyl (NIDA, 2021). The influence of biological factors, such as sex, on risks and outcomes of OUD are important to consider given that the opioid epidemic has been particularly impactful on women. For example, although men have higher rates of OUD and opioid-induced overdose deaths than women, differences have narrowed in the current opioid epidemic (i.e., 2.3:1 male-to-female ratio in 2002 versus a 1.8:1 ratio in 2018; National Survey on Drug Use and Health, 2018) with women showing a sharper increase in opioid use in the past decade than men (e.g., 283% versus 108% increase in heroin use from 2007 to 2014; Marsh et al., 2018) and being more likely than men to be prescribed opioids and to misuse prescription opioids (Mazure and Fiellin, 2018). Women are also more sensitive to the reinforcing effects of opioids, develop OUD more rapidly, and have higher cravings in response to drug cues compared with men (Anglin et al., 1987; Hser et al., 1987; Lynch and Carroll, 2001; Hernandez-Avila et al., 2004; Yu et al., 2007; Back et al., 2011a; Kennedy et al., 2013; Adelson et al., 2018; Moran et al., 2018).

In response to this need to understand the impact of biological sex on the risks and outcomes of OUD with fentanyl, we recently developed an extended-access fentanyl self-administration procedure that readily induces addiction-like features, including binge-abstinent patterns of use and an enhanced vulnerability to relapse, in both male and female rats (Bakhti-Suroosh et al., 2021). This latter feature, the enhanced vulnerability to relapse, emerged following extended-access self-administration and protracted withdrawal, and was blocked in both males and females by administering buprenorphine, an FDA-approved treatment for OUD, during abstinence, thus, validating our relapse model. Importantly, our preclinical findings were also similar to reports of sex/gender differences in humans and showed that females self-administered higher levels of fentanyl during the extended-access phase and responded at higher levels than males during subsequent relapse testing, especially when they were tested during estrus versus non-estrus phases of their cycles. These findings demonstrated that both males and females developed an addiction-like phenotype when given extended access to fentanyl and demonstrated that sex is an important risk factor for both intake and the development of expression of relapse vulnerability with fentanyl.

Now in this study, our goal was to determine sex differences across a broad dose range, including high doses expected to maximize the expression of addiction-like features (e.g., vulnerability to relapse and physical dependence). This is important because in our original study, we focused on the effects of a low dose of fentanyl (0.25 μg/kg/infusion) since low doses engender greater individual differences and are, thus, more sensitive to sex differences. However, low doses may not maximally induce an addiction-like phenotype considering that higher drug intake and/or frequency of use is predictive of an enhanced vulnerability to relapse in both humans with an OUD (Gossop et al., 2002; Smyth et al., 2010; Grau-López et al., 2012) and in animal models with other addictive drugs (Mantsch et al., 2004). Thus, in the current study, we examined fentanyl self-administration across a broad range of fentanyl doses (0.25, 0.75, 1.5, and 3.0 µg/kg/infusion) and hypothesized that relapse vulnerability would be highest following high-dose fentanyl self-administration. We also expanded our model to include an additional key feature of OUD in humans, physical dependence (American Psychiatric Association, 2013), as assessed by spontaneous weight loss during early withdrawal, a highly predictive single factor of withdrawal (Cicero and Meyer, 1973; Gellert and Holtzman, 1978; Nickel and Aledter, 1987; Maldonado et al., 1992; Houshyar et al., 2004; Navarro-Zaragoza et al., 2010; Pintér-Kübler et al., 2013; Bobzean et al., 2019; Seaman and Collins, 2021; Townsend et al., 2021). Physical dependence is a defining feature of OUD in humans (American Psychiatric Association, 2013), and women experience a more severe withdrawal syndrome than men (Huhn and Dunn 2020). Given that higher drug intake/frequency of use is also predictive of greater physical dependence, we hypothesized that weight loss would be greatest following high-dose fentanyl self-administration. Based on findings in humans and our previous results with fentanyl, we further hypothesized that the expression of enhanced vulnerability to relapse would be greater in females than males.

## Methods

### Subjects

Sexually mature male (N = 29) and female (N = 29) Sprague–Dawley rats (Charles River) that weighed approximately 250 g (female) and 340 g (male) upon arrival were used as subjects in this study. At the start of the study, rats were individually housed in operant test chambers (Med Associates, St. Albans, VT, USA) with *ad libitum* access to water and food (Teklad LM-485 7912; except as noted below for some animals during fentanyl self-administration training) and maintained on a 12-h light/dark cycle (lights on at 7a.m.). After a 2-day acclimation period, rats were pretrained to lever press for sucrose pellets (45 mg) in 24-h/day sessions under a fixed-ratio 1 schedule to ensure rapid subsequent acquisition of fentanyl self-administration. Sessions continued daily until lever-press responding was acquired (two consecutive days wherein >50 pellets were obtained, typically two to three sessions; Lynch, 2008). Rats were weighed three times a week, and health was monitored daily throughout the study. Body weight was used as an assessment of overall health during extended access and as a measure of physical dependence to fentanyl. Physical dependence has been assessed previously by expression of opioid withdrawal syndrome upon cessation of chronic opioid exposure, and spontaneous loss of body weight has long been used as a highly predictive single factor of withdrawal (Cicero and Meyer, 1973; Gellert and Holtzman, 1978; Nickel and Aledter, 1987; Maldonado et al., 1992; Houshyar et al., 2004; Navarro-Zaragoza et al., 2010; Pintér-Kübler et al., 2013). All procedures were conducted within the animal care guidelines set by the National Institute of Health and were approved by The University of Virginia Animal Care and Use Committee.

### Procedure

#### Surgery and catheter maintenance

After lever pretraining, rats underwent jugular catheterization surgery using methods previously described (Lynch 2008). Briefly, rats were anesthetized with ketamine/dexdomitor and implanted with an indwelling catheter (Silastic tubing; 0.51 and 0.94 mm o.d.; Dow Corning, Midland, MI, USA) into the right jugular vein. Catheters were flushed with heparinized saline 3 days a week to help verify and help maintain patency. If the patency of a catheter was questionable, patency was verified by administering methohexital (1.5 mg/kg). Any catheter that was no longer patent (i.e., the catheter was leaking, pressure prevented flushing, or the animal did not lose the righting reflex immediately after methohexital) was replaced with a new catheter implanted into the left jugular vein with testing resuming following recovery from surgery (1–2 days).

#### Fentanyl self-administration training

Following recovery from surgery, rats were randomly assigned to self-administer one of four fentanyl doses (µg/kg/infusion): 0.25 (9 females and 8 males), 0.75 (9 females and 8 males), 1.5 (8 females and 8 males), or 3.0 (7 females and 8 males). These doses were selected because the majority of studies using fentanyl self-administration procedures in rodents use a dose of fentanyl ranging from 0.25 to 2.5 ug/kg (Morgan et al., 2002; Wade et al., 2015; Bakhti-Suroosh et al., 2021; Dao et al., 2021; Fragale et al., 2021; Hammerslag et al., 2021; Malone et al., 2021; Martin et al., 2021); therefore, we selected a dose range that included both low (0.25 and 0.75 µg/kg/infusion) and moderate-to-high doses (1.5 and 3.0 µg/kg/infusion; Morgan et al., 2002; Wade et al., 2015; Martin et al., 2021; Dao et al., 2021; Malone et al., 2021; Hammerslag et al., 2021) in order to maximize the likelihood of sex and group differences in levels and patterns of fentanyl self-administration and subsequent effects on relapse vulnerability. Rats were trained to self-administer their assigned dose of fentanyl under a fixed-ratio 1 schedule with a 1-s time out following each infusion and a maximum of 40 infusions/day (Bakhti-Suroosh et al., 2021). At the beginning of each session, the left lever was extended into the chamber and remained extended until the session ended once all 40 infusions were obtained or until 11:50 a.m. the next day. Each response on the left lever produced an infusion of fentanyl, which was paired with the sound of the pump and the illumination of a stimulus light above the lever. The right lever remained extended throughout the session, and responses on this lever (inactive) were recorded but had no consequence. Sessions were conducted daily until acquisition occurred (i.e., 5 consecutive days wherein all 40 infusions were obtained). Moderate food restriction (85% of its free-feeding body weight) was used briefly (2–3 days) when necessary (i.e., fewer than 15 infusions/day by training day 5). All groups acquired fentanyl self-administration rapidly under these conditions, and rates of acquisition did not differ between groups.

#### Extended-access fentanyl self-administration

Once rats acquired fentanyl self-administration, they were given extended, 24 h/day access to fentanyl for 10 consecutive days under an intermittent-access procedure shown to induce an addiction-like phenotype in both males and females (Bakhti-Suroosh et al., 2021). With this procedure, rats have unrestricted, fixed-ratio 1 access (no timeout after infusions) to rapidly delivered infusions of fentanyl (within 1–2 s) during 5-min trials that initiated every 30 min around the clock. Each trial began with the extension of the left lever into the operant chamber; each response on this lever resulted in an infusion of fentanyl paired with the sound of the pump and the illumination of the stimulus light above the active lever. The 5-min trial ended with the left lever being retracted from the operant chamber. The right lever remained extended for the entire duration of the session; responses on this lever were recorded but had no consequence. Two females in the 0.25 μg/kg group, one female and one male in the 0.75 μg/kg group, and one female and one male in the 1.5 μg/kg group were excluded from the study and all analyses due to patency, toxicity, or technical issues during acquisition or extended-access self-administration. The final group sizes for females and males were 7 and 7 for the 0.25 μg/kg group, 8 and 7 for the 0.75 μg/kg group, 7 and 7 for the 1.5 μg/kg group, and 7 and 8 for the 3.0 μg/kg group, respectively.

#### Extinction and reinstatement testing

Vulnerability to relapse was assessed on withdrawal day15 using an extinction/cue-induced reinstatement procedure (Bakhti-Suroosh et al., 2021). Testing began between 12 and 1 p.m. with extinction responding being examined in a minimum of six 1-h sessions (Sanchez et al., 2014; Peterson et al., 2014; Beiter et al., 2016). Each session began with the introduction of the left lever into the operant chamber; responses on this lever, as well as the right lever, were recorded but did not have a consequence. Sessions continued until responding was extinguished (≤15 responses/h). This extinction criterion was typically met within six to nine sessions and with the exception of two males and three females (as detailed below). Cue-induced reinstatement responding was assessed 5 min after the final extinction session in a 1-h session. This session began with the introduction of the left lever into the operant chamber and the presentation of the cues formerly associated with fentanyl (sound of pump activation and the light above the left lever, 1–2 s). Each response on the left lever produced these same cues under a fixed-ratio 1 schedule. For the two males (one each in the 0.75 and the 1.5 μg/kg dose groups) and three females (two in the 0.25 μg/kg dose group and one in 1.5 μg/kg dose group) that did not extinguish within the nine extinction sessions run, their sessions terminated following the ninth extinction sessions, and then the next day, a second day of extinction testing was conducted using the same procedures (i.e., six to nine 1-h sessions) to ensure that responding was extinguished prior to reinstatement testing, and reinstatement testing was conducted during a similar time in the light cycle. The reinstatement test session began once responding had extinguished using the same procedure as described above. Data from the first day of extinction testing were used in the analyses of hourly extinction responses, whereas the second day was used for the last extinction session (versus reinstatement).

#### Estrous cycle phase determination

In order to track the pattern of the estrous cycle leading up to relapse testing and to habituate rats to the procedure, the phase of the estrous cycle was determined daily over a 5-day period beginning 3 days prior to extinction/reinstatement testing. The swabs of the vaginal epithelium cells were collected between 11 a.m. and 12 p.m.; male rats underwent similar handling by brushing their rear end with the cotton swab as described previously (Lynch et al., 2019). The phase of the estrous cycle was determined based on the proportion of three vaginal cell types: leukocytes, nucleated epithelial cells, and cornified epithelial cells. The rat was considered to be in estrus if there were an abundant number of cornified epithelial cells with no leucocytes, metestrus or diestrus if leukocytes were present, and proestrus if there were numerous, uniform in size round nucleated cells and no or few leucocytes. Swabs obtained on the day of extinction/reinstatement test were further categorized as either estrus (*n* = 13) or non-estrus (*n* = 17) based on findings from our group (Peterson et al., 2014; Lynch et al., 2019) and others (Kerstetter et al., 2008) showing that relapse vulnerability, including opioid seeking, is highest during estrus, but not different between metestrus, diestrus, and proestrus (Lynch et al., 2019; Nicolas et al., 2019; Lacy et al., 2020; Bakhti-Suroosh et al., 2021; Corbett et al., 2021).

### Drugs

Fentanyl hydrochloride was obtained from the National Institute on Drug Abuse (Research Triangle Park, NC, USA) and dissolved in sterile saline at a concentration of 6.25, 18.75, 37.5, or 75 µg/ml for the 0.25, 0.75, 1.5, and 3.0 µg/kg dose conditions, respectively. Fentanyl solutions were sterile filtered (0.22 μm; Millipore, Billerica, MA, USA) and stored at 4°C. The duration of infusions was adjusted for changes in body weight on Mondays, Wednesdays, and Fridays to ensure that the mg/kg dose was consistent throughout the study.

### Analysis

We first determined whether there was an effect of fentanyl dose on levels (μg/kg/day) and patterns of intake over the 10day extended-access self-administration period. Patterns of intake included frequency of use (total number of infusions/day and total intake/day in µg/kg), number of active trials within each extended-access session, and “binge” intake (average intake/day in µg/kg within each of the “active” trials that had one or more infusions). Group differences were assessed using repeated measures ANOVA with sex and fentanyl dose as between-subject factors and extended-access session as a within-subject factor; separate analyses were used for each of the dependent measures. Given that sex differences have previously been shown to be most robust under low versus high drug dose conditions (Lynch and Carroll 2001; Carroll et al., 2004; Torres et al., 2014; Towers et al., 2019), following a significant interaction of sex by dose, we examined sex differences within the two low fentanyl dose groups (0.25 and 0.75 μg/kg) and the two high dose groups (1.5 and 3.0 μg/kg). Repeated measures ANOVA was also used to examine sex and group differences in body weight over arrival, training (days 3–5), extended access (ExA; ExA 1, days 4–6; and ExA 2, days 9, 10, and first day of withdrawal at the time of discontinuation of drug self-administration), and withdrawal (W1, days 2–4; W2, days 6–8; and W3, days 12–14). To equate baseline sex differences in body weight, the same analysis was ran for percent change in body weight over ExA-1 and ExA-2 relative to prior to the beginning of extended access (training) as an assessment of overall health, and percent change in body weight over W1, W2, and W3 relative to ExA-2 as a measure of physical dependence to fentanyl. This measure (e.g., weight loss during spontaneous withdrawal) is a known proxy of physical dependence in animals and has long been used as a highly predictive and single factor of withdrawal (Cicero and Meyer, 1973; Gellert and Holtzman, 1978; Nickel and Aledter, 1987; Maldonado et al., 1992; Houshyar et al., 2004; Navarro-Zaragoza et al., 2010; Pintér-Kübler et al., 2013). A one-sample *t*-test was used to confirm significant decrease or increase in changes of body weight relative to baseline (or 0).

Effects on extinction and reinstatement were compared between the sexes and each of the dose groups as well as between males and females tested during estrus versus non-estrus phases. For extinction, we compared hourly responses on the formerly active lever within the first six extinction sessions run using repeated measures. The total number of responses on the formerly active lever were also compared using univariate ANOVA. For reinstatement, we compared responses on the formerly active lever during the last extinction session versus the cue-induced reinstatement session. Associations between fentanyl intake/infusions and total extinction and reinstatement responding/weight loss during withdrawal were assessed using the Pearson correlation coefficient. The analysis was performed collapsed across sex unless the univariant ANOVA determined there was a significant difference in the correlation coefficients for males and females. All *post hoc* comparisons were corrected for multiple comparisons using Tukey’s method. One-tailed tests were used for all *a priori* predicted differences (higher intake/infusions in females than males, greater fentanyl seeking in estrus females versus males and non-estrus females, positive association between fentanyl intake/infusions and extinction/reinstatement responding); all other tests were two tailed. Statistical analyses were performed using SPSS (V26) with alpha set at 0.05.

## Results

### Extended-access fentanyl self-administration

**FIGURE 1:**
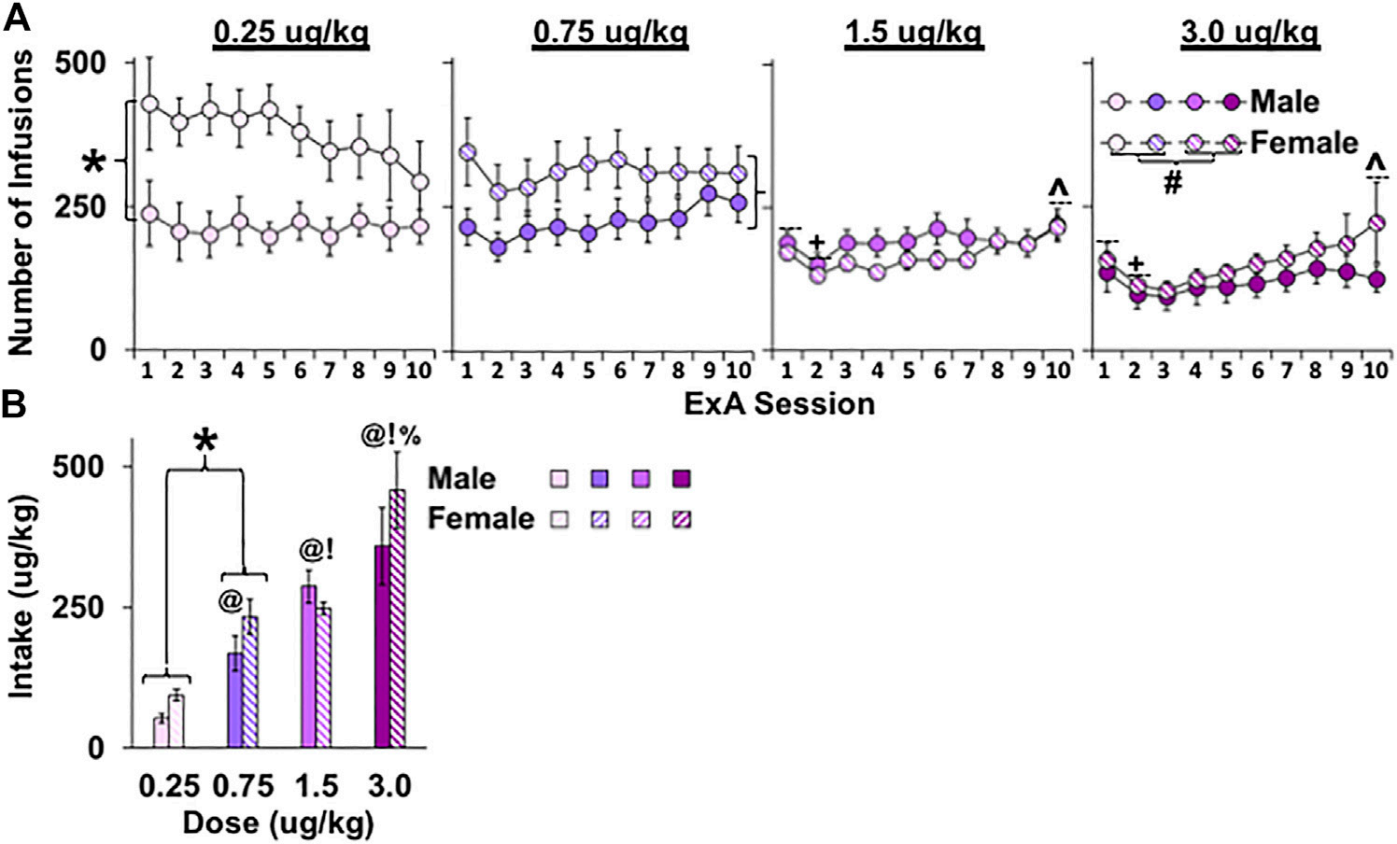
Effect of sex and dose on the number of infusions and fentanyl intake in female and male rats under extended-access conditions. Mean (±SEM) number of infusions for each of the 10 extended-access sessions **(A)** and fentanyl intake averaged across the extended-access period (μg/kg); **(B)** for females and males in the 0.25 (*n* = 8 males, *n* = 9 females), 0.75 (*n* = 8 males, *n* = 9 females), 1.5 (*n* = 8 males, *n* = 8 females), and 3.0 (*n* = 8 males, *n* = 7 females) dose (μg/kg) conditions. (*) Significant sex difference within the two lower doses. (+) Significant decrease from session 1. (^) Significant increase from session 2. (#) Significant difference between the two lowest and two highest doses in females. Significant difference from (@) 0.25, (!) 0.75, and (%) 1.5 μg/kg.

Sex and dose-dependent effects were observed for the number of infusions self-administered over the 10-day extended-access period (Figure 1A) with results revealing significant overall effects of sex (F_1, 50_ = 8.5, *p* < 0.01), dose (F_3, 50_ = 11.4, *p* < 0.001), and session (F_9, 450_ = 2.6, *p* < 0.01) as well as significant interactions of sex by dose (F_3, 50_ = 3.2, *p* < 0.05) and session by dose (F_27, 450_ = 1.6, *p* < 0.05). While the overall effect of sex indicates higher infusions in females than in males, this difference appears to be driven primarily by effects at the lower doses given the sex by dose interaction as well as results from the subsequent analyses within the low (0.25 and 0.75 μg/kg) and high (1.5 and 3.0 μg/kg) doses, which revealed a significant effect of sex within the low (F_1, 27_ = 9.7, *p* < 0.01), but not high, doses (*p* > 0.05). While the overall effect of dose reflects higher infusions at the lower versus higher doses (0.25 versus 1.5 and 3.0 μg/kg, *p*’s <0.05; 0.75 versus 1.5 and 3.0 μg/kg; *p*’s < 0.05), this difference appears to be driven primarily by effects in females given the significant interaction of sex by dose as well as results from the follow-up comparisons within females and males, which revealed a significant effect of dose within females (F_3, 25_ = 11.4, *p* < 0.001), but only a trend for an effect of dose within males (*p* = 0.076). Further comparison within females revealed significant differences between the two lower doses and the two higher ones (0.25 versus 1.5 and 3.0 μg/kg, *p*’s < 0.001; 0.75 versus 1.5 and 3.0 μg/kg, *p*’s < 0.05). Finally, the overall effect of session appears to be attributable to a decrease in infusions from sessions 1 to 2 (t_57_ = 4.2, *p* < 0.001) as well as an increase in infusions from session 2 to 10 (t_57_ = 2.3, *p* < 0.05). However, both effects were apparent for the two highest doses, but not the two lowest doses, which likely account for the significant interaction observed between dose and session. Indeed, subsequent analysis within each of the doses revealed a significant overall effect of session within the 1.5 and 3.0-μg/kg doses (F_9, 108_ = 4.1, *p* < 0.001 and F_9, 117_ = 3.7, *p* < 0.001, respectively), but not the 0.25 or 0.75-μg/kg doses (*p*’s > 0.05); subsequent analyses within the 1.5 and 3.0-μg/kg doses also confirmed a significant decrease in infusions from sessions 1 to 2 and a significant increase in infusions from sessions 2 to 10 for both doses (*p*’s < 0.01). Thus, females self-administered more fentanyl infusions than males, particularly at low doses.

Average daily fentanyl intake (µg/kg) over the extended-access period was greatest in rats given access to higher versus lower doses of fentanyl (Figure 1B; effect of dose, F_3, 50_ = 23.4, *p* < 0.001) with the 3.0 μg/kg group having the highest intake (versus 0.25, 0.75, and 1.5 μg/kg, *p* < 0.05) and the 0.25 μg/kg group having the lowest intake (versus 0.75 and 1.5 μg/kg, *p*’s < 0.05). In contrast to effects with infusions, dose-dependent effects on intake were apparent for both males and females (nonsignificant interaction of dose by sex). Although no overall or interactive effects of sex were observed for intake (*p*’s > 0.05), a planned comparison of males and females in the low-dose groups (0.25 and 0.75 μg/kg) confirmed that, similar to the effects with infusions, females had higher fentanyl intake than males (t_27_ = 1.7, *p* < 0.05). Thus, fentanyl intake dose-dependently increased in both males and females with increases in fentanyl dose with females taking more fentanyl than males at low fentanyl doses.

**FIGURE 2:**
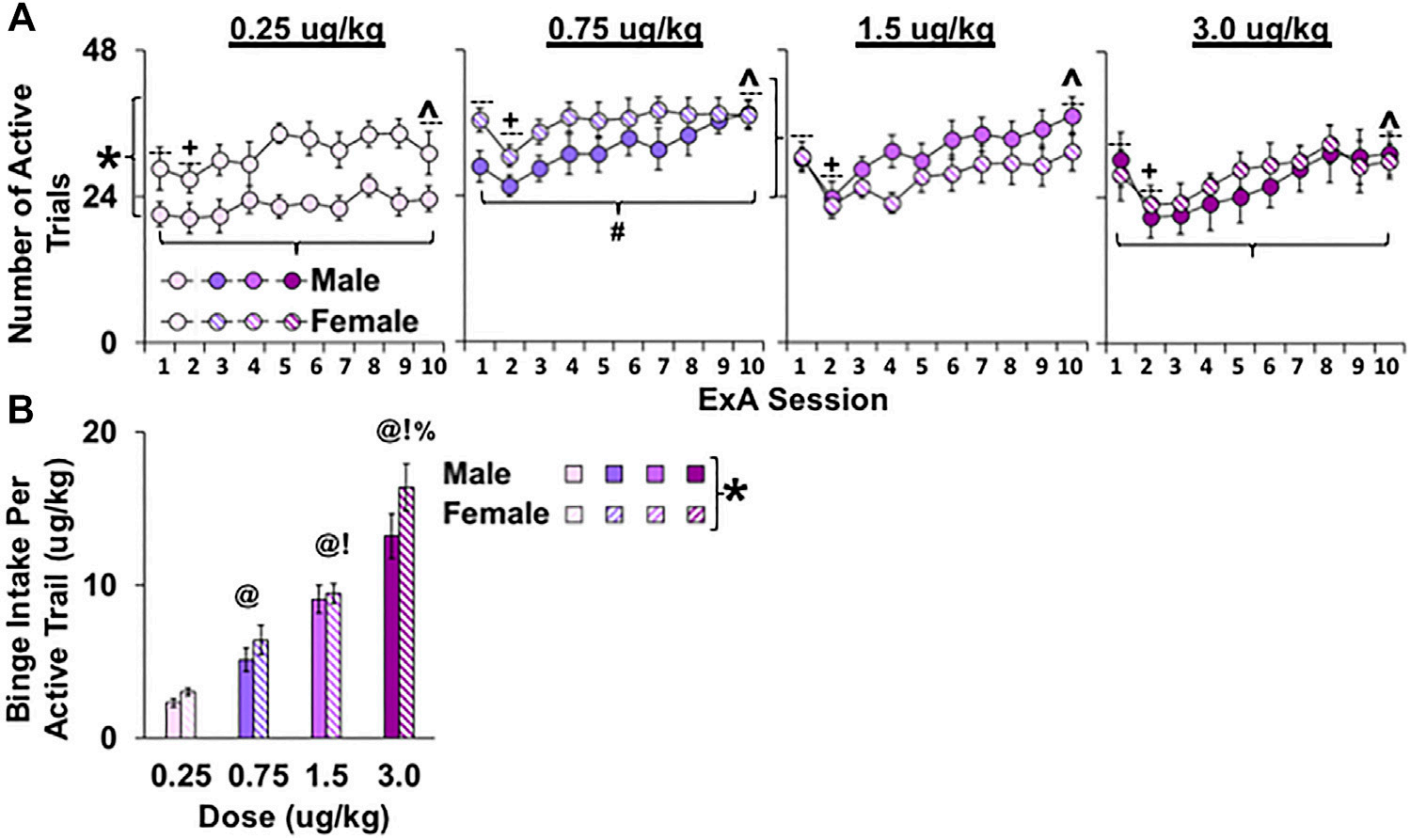
Effect of sex and dose on binge fentanyl intake in female and male rats under extended-access conditions. Mean (±SEM) number of active trials for each of the 10 extended-access sessions **(A)** and binge fentanyl intake (μg/kg) in active trials across all extended-access session **(B)** for females and males within the 0.25 (*n* = 8 males, *n* = 9 females), 0.75 (*n* = 8 males, *n* = 9 females), 1.5 (*n* = 8 males, *n* = 8 females), and 3.0 (*n* = 8 males, *n* = 7 females) dose (μg/kg) conditions. (*) Significant sex difference within the two lower doses. (+) Significant decrease from session 1. (^) Significant increase from session 2. (#) Significant increase compared with 0.25 and 3.0 μg/kg. Significant difference from (@) 0.25, (!) 0.75, and (%) 1.5 μg/kg.

To further explore sex and dose-dependent differences in patterns of fentanyl self-administration, we also analyzed the number of active trials during each of the 10 extended-access sessions. This analysis revealed an overall effect of dose (Figure 2A; F_3, 50_ = 3.7, *p* < 0.05) and session (F_9, 450_ = 21.0, *p* < 0.001) and a trend for an interaction of sex by dose (*p* = 0.069) and session by dose (*p* = 0.085). The overall effect of dose appears to be attributable to rats in the 0.75-μg/kg dose having significantly more active trials compared with the lowest and highest dose conditions (0.75 versus 0.25 and 3.0 μg/kg, *p*’s < 0.05). As with findings for daily intake, the session effect in this analysis appears to be due to a decrease in the number of active trials from sessions 1 to 2 (t_57_ = 6.4, *p* < 0.001) and an increase from sessions 2 to 10 (t_57_ = 8.4, *p* < 0.001). However, these session effects appear to be more robust at higher versus lower doses, which likely accounts for the trend for an interaction between dose and session. Although no overall effect of sex was observed (*p* > 0.05), given the trend for an interaction of sex by dose (*p* = 0.069) and our hypothesis that sex differences would be most apparent under low dose conditions, we examined sex differences within the low versus high dose groups. This analysis showed, that as with daily intake, females had more active fentanyl trials than males under low (F_1, 27_ = 7.9, *p* < 0.01), but not high dose (*p* > 0.05) conditions. Thus, rats in the 0.75 μg/kg group had more active trials than rats in the other dose groups, and females in the low dose groups had more active trials than males in these groups.

Average binge fentanyl intake (µg/kg) within active trials across the 10 extended-access sessions was greater in females compared with males (Figure 2B; overall effect of sex, F_1, 50_ = 4.8, *p* < 0.05) and in higher versus lower dose conditions (F_3, 50_ = 61.4, *p* < 0.001; 3.0 versus 0.25, 0.75, and 1.5 μg/kg, *p*’s < 0.010; 1.5 versus 0.25 and 0.75, *p*’s < 0.01; and 0.75 versus 0.25, *p* < 0.01). Given that there were no significant overall or interactive effects of session (*p*’s > 0.05), these data are presented as average binge intake/day across the extended-access period to highlight the overall effects of sex and dose. There were no interactions of dose or sex (*p*’s > 0.05). Thus, in contrast to daily intake, the higher binge intake in females was similarly maintained across low and high fentanyl doses. However, similar to daily fentanyl intake, binge intake increased in both sexes with increases in fentanyl dose.

### Changes in body weight during extended-access self-administration and over withdrawal

**FIGURE 3:**
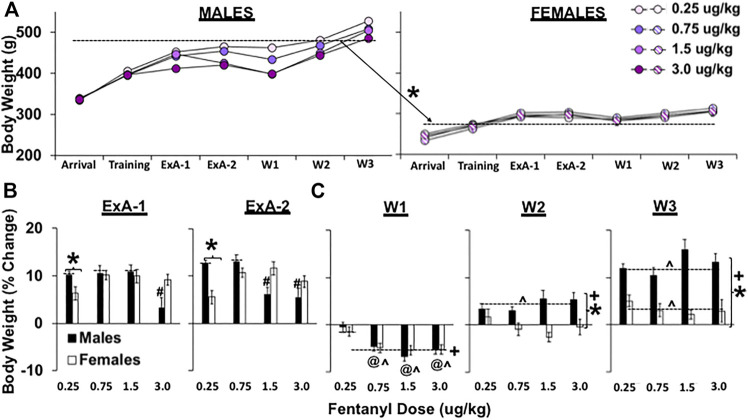
Effect of sex and dose on body weight (g) in female and male rats during extended-access self-administration and withdrawal. Mean (±SEM) body weight (g) at arrival, the end of self-administration training (training, days 3–5) just prior to extended-access self-administration, following approximately 5 days (ExA-1, days 4–6) and 10 days (ExA-2, days 9–10 or at the time of discontinuation of drug self-administration on withdrawal day 1) of extended-access self-administration, and during early (W1, days 2–4), intermediate (W2, days 6–9), and late withdrawal (W3, days 12, 13, and 14) for males and females in the 0.25 (*n* = 8 males, *n* = 9 females), 0.75 (*n* = 8 males, *n* = 9 females), 1.5 (*n* = 8 males, *n* = 8 females), and 3.0 (*n* = 8 males, *n* = 7 females) dose (μg/kg) conditions **(A)**. These data are also plotted as percent change in body weight after approximately 5 (ExA-1) and 10 (ExA-2) days of extended-access self-administration relative to the end of training (training) just prior to extended-access self-administration **(B)** and during early, intermediate, and late withdrawal relative to the end of extended-access self-administration (ExA-2) just prior to withdrawal **(C)**. (*) Significant effect of sex. (#) Significant difference from higher doses in males. (+) Significant difference between each of the withdrawal phases. (@) Significant difference from 0.25 μg/kg. (^) Significant difference from prewithdrawal body weight (versus 0 or no change).

As expected, there was a marked sex difference in body weight across the study (Figure 3A; overall effect of sex, F_1, 50_ = 1338.6, *p* < 0.001) with males weighing more than females. There were also significant overall effects of dose (F_1, 50_ = 4.2, *p* = 0.01) and time (F_6, 300_ = 508.2, *p* < 0.001), and interactions of time by sex (F_6, 300_ = 95.7, *p* < 0.001), time by dose (F_18, 300_ = 3.3, *p* < 0.001), and time by sex by fentanyl dose (F_18, 300_ = 4.3, *p* < 0.001). Further analysis of body weight at the end of fentanyl self-administration training (days 3–5) just prior to the start of extended access confirmed an overall effect of sex (F_1, 50_ = 906.7, *p* < 0.001) with males weighing more than females (*p* < 0.001), but no overall or interactive effects of dose (*p*’s > 0.05); therefore, to determine sex- and dose-dependent effects of extended-access fentanyl self-administration on body weight, we analyzed percent change in body weight from just prior to extended-access self-administration (training) to after approximately 5 (ExA-1) or 10 (ExA-2) days of access (Figure 3B). Results from this analysis revealed an overall effect of dose (F_3, 50_ = 4.0, *p* < 0.05) as well as significant interactions of sex by dose (F_3, 50_ = 5.5, *p* < 0.01) and sex by day by dose (F_3, 50_ = 3.9, *p* < 0.05). While the overall effect of dose reflects lower percent body weight gain in the 3.0 μg/kg group compared with the 0.75 μg/kg group (*p* < 0.05), this difference appears to be driven by males given the significant interaction between dose and sex and the follow-up comparisons within males and females, which revealed a significant effect of dose within males (F_3, 25_ = 7.7, *p* < 0.001), but only a trend for an effect of dose within females (*p* = 0.084). The analysis within males also revealed a significant interaction of day by dose (F_3, 25_ = 32.7, *p* < 0.05) with follow-up comparisons within ExA-1 and 2 revealing that males in the 3.0 μg/kg dose gained significantly less weight compared with males in the 0.25, 0.75, and 1.5 μg/kg doses at ExA-1 and that males in both the 1.5 and 3.0 μg/kg doses gained significantly less weight than males in the 0.25 and 0.75 μg/kg dose at ExA-2 (*p*’s < 0.05). Given the significant interactions of sex by dose and sex by dose by day, we also examined sex differences within each of the dose groups. This analysis revealed a significant effect of sex within the 0.25-μg/kg dose group (F_1, 12_ = 12.0, *p* < 0.01), wherein females had less percent body weight gain than males, as well as a trend for an effect of sex in the 3.0 dose group (*p* = 0.052), wherein males tended to have less percent body weight gain than females. Thus, in males, fentanyl dose-dependently decreased percent body weight gain and the highest dose tended to have a greater anorectic effect in males than in females. In contrast, females showed an enhanced sensitivity to the anorectic effect of the low dose of fentanyl compared with males, and although this effect may be the result of greater fentanyl intake in females than in males at the lower doses, it was not further enhanced in females with increases in fentanyl dose.

We also analyzed percent change in body weight during early (W1, withdrawal days 2–4), intermediate (W2, withdrawal days 6–8), and late withdrawal (W3, withdrawal days 13–15) relative to the end of the extended-access self-administration (ExA-2) as a measure of physical dependence to fentanyl (Figure 3C). This analysis revealed significant overall effects of sex (F_2, 50_ = 33.2, *p* < 0.001) and withdrawal timepoint (early, immediate, and late; F_2, 100_ = 341.4, *p* < 0.001), which reflect greater increases in percent body weight in males versus females and at later versus earlier timepoints during withdrawal (early versus intermediate and late, *p*’s < 0.001; intermediate versus late, *p* < 0.05) as well as significant interactions of sex by withdrawal timepoint (F_2, 100_ = 50.5, *p* < 0.001) and sex by withdrawal timepoint by dose (F_6, 100_ = 2.7, *p* < 0.05). The overall sex effect appears to be driven by changes during intermediate and late withdrawal given the significant interaction of sex by withdrawal timepoint and the results from analysis within each withdrawal timepoint, which revealed significant effects of sex within the intermediate and late withdrawal timepoints (*p*’s < 0.001), but not within the early withdrawal timepoint (*p* > 0.05). Further analysis within the early withdrawal timepoint revealed a significant effect of dose (F_3, 50_ = 7.9, *p* < 0.001) and significant differences between the 0.25 μg/kg dose and all other doses (*p*’s < 0.05). The decreases in percent body weights during early withdrawal were also significantly different from body weights at the end of the extended access (ExA-2; versus 0) for each of the doses except the 0.25 μg/kg dose (*p*’s < 0.001). Further analysis within intermediate and late withdrawal revealed nonsignificant overall and interactive effects of dose indicating that the sex differences were due to greater percent body weight gain in males than females in each of the dose groups with males, but not females, surpassing their previous body weight at ExA2 by intermediate withdrawal (*p* < 0.001); by late withdrawal, both males and females had surpassed their previous body weight at ExA-2 (versus 0; *p*’s < 0.001). Thus, rats in the three highest dose groups lost body weight during early withdrawal and this weight loss was similar between males and females indicating that physical dependence was expressed similarly in males and females following fentanyl self-administration at 0.75 μg/kg doses and higher. Despite the similar weight loss between males and females during early withdrawal, weight loss persisted longer in females compared with males.

### Extinction and reinstatement of fentanyl seeking

**FIGURE 4:**
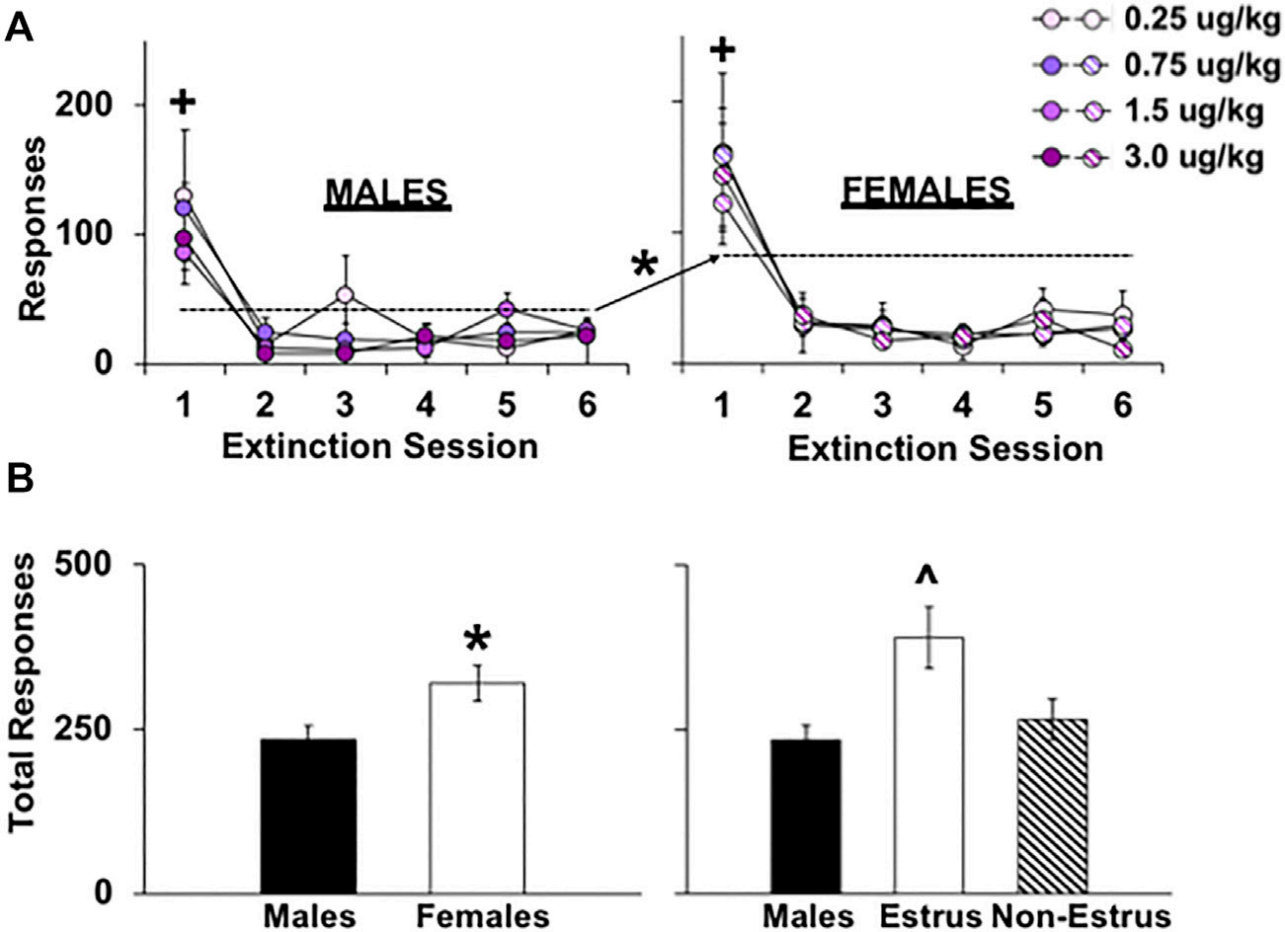
Effect of sex, estrous cycle phase, and dose on responding during extinction testing in female and male rats. Mean (±SEM) number of responses made on the lever formerly associated with fentanyl during the first six 1-h extinction sessions for males **(A; left)** and females (right) within the 0.25 (*n* = 8 males, *n* = 9 females), 0.75 (*n* = 8 males, *n* = 9 females), 1.5 (*n* = 8 males, *n* = 8 females), and 3.0 (*n* = 8 males, *n* = 7 females) dose (μg/kg) conditions and across all doses and extinction sessions run **(B)** (*n* = 29 males, *n* = 29 females). (*) Significantly higher responding in females compared with males. (+) Significantly higher responding in session 1 compared with sessions 2–6. (^) Significantly higher responding in estrus females compared with non-estrus females and males.

To our surprise, the dose of fentanyl self-administered during the extended-access period did not impact extinction responding over the first six extinction sessions (Figure 4A; no overall or interactive effects of dose, *p*’s > 0.05). As expected, however, females responded at higher levels than males (effect of sex, F_1, 250_ = 4.9, *p* < 0.05). Responding was also highest during the first extinction session compared with the later ones (effect of session, F_5, 250_ = 40.7, *p* < 0.001; session 1 compared with sessions 2–6, *p* < 0.001). Analysis of total extinction responding confirmed the nonsignificant overall and interactive effect of dose (*p*’s > 0.05) and higher responses in females than males (F_1, 50_ = 5.3, *p* < 0.05; Figure 4B). Extinction responding also differed between males and females tested during estrus versus non-estrus phases (F_1, 46_ = 6.3, *p* < 0.01) with estrus females responding at higher levels compared with both males (*p* < 0.001) and non-estrus females (*p* < 0.05).

**FIGURE 5:**
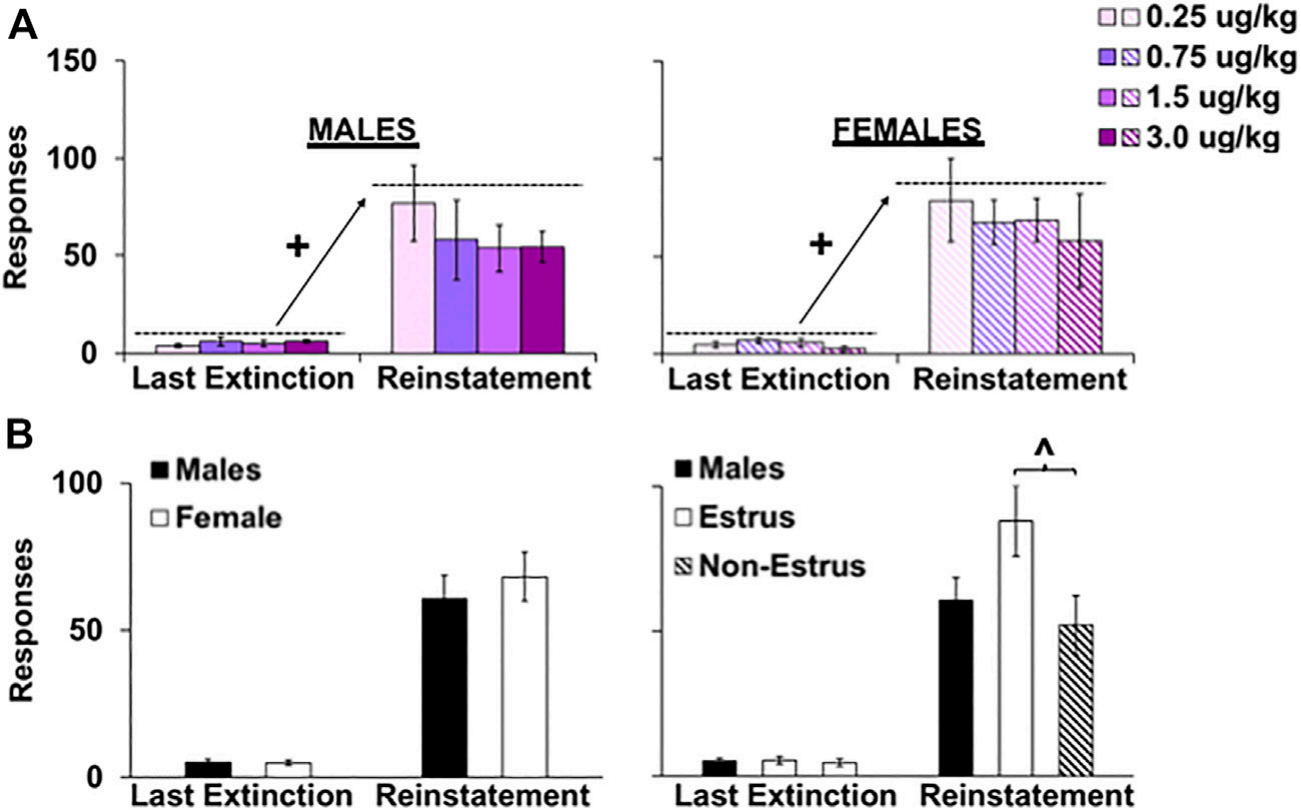
Effect of sex, estrous cycle phase, and dose on responding during reinstatement testing in female and male rats. Mean (±SEM) number of responses made on the lever formerly associated with fentanyl during the last extinction session versus the reinstatement session for males **(A; left)** and females (right) within the 0.25 (*n* = 8 males, *n* = 9 females), 0.75 (*n* = 8 males, *n* = 9 females), 1.5 (*n* = 8 males, *n* = 8 females), and 3.0 (*n* = 8 males, *n* = 7 females) dose (μg/kg) conditions and across all doses **(B)** (*n* = 29 males, *n* = 29 females). (+) Significantly higher responding in the reinstatement session compared with the last extinction session. (^) Significantly higher responding in estrus females compared with non-estrus females.

Similar to the effects during extinction, the dose of fentanyl self-administered during the extended-access period did not impact cue-induced reinstatement responding (Figure 5A). Specifically, results from the repeated measures ANOVA comparing responding during the last extinction session to the reinstatement session revealed a significant overall effect of session (F_1, 50_ = 100.1, *p* < 0.001), but nonsignificant overall or interactive effects of dose (*p*’s > 0.05) indicating that fentanyl-seeking was similarly reinstated within each of the fentanyl groups. In contrast to the extinction findings, there was also no overall or interactive effect of sex (Figure 5B; *p* > 0.05). However, as predicted, reinstatement responding was higher in females tested during estrus versus non-estrus phases (*p* 0.05); estrus females also tended to respond at higher levels than males (*p* = 0.08). Inactive lever responses during extinction and reinstatement were minimal and analysis of inactive lever responses revealed no overall or interactive effects of sex or groups (*p* > 0.05). Thus, the dose of fentanyl self-administered during extended access did not have a significant effect on subsequent relapse vulnerability; relapse vulnerability was most pronounced in females during estrus.

### Associations between frequency or amount of intake and vulnerability to relapse or physical dependence

**FIGURE 6:**
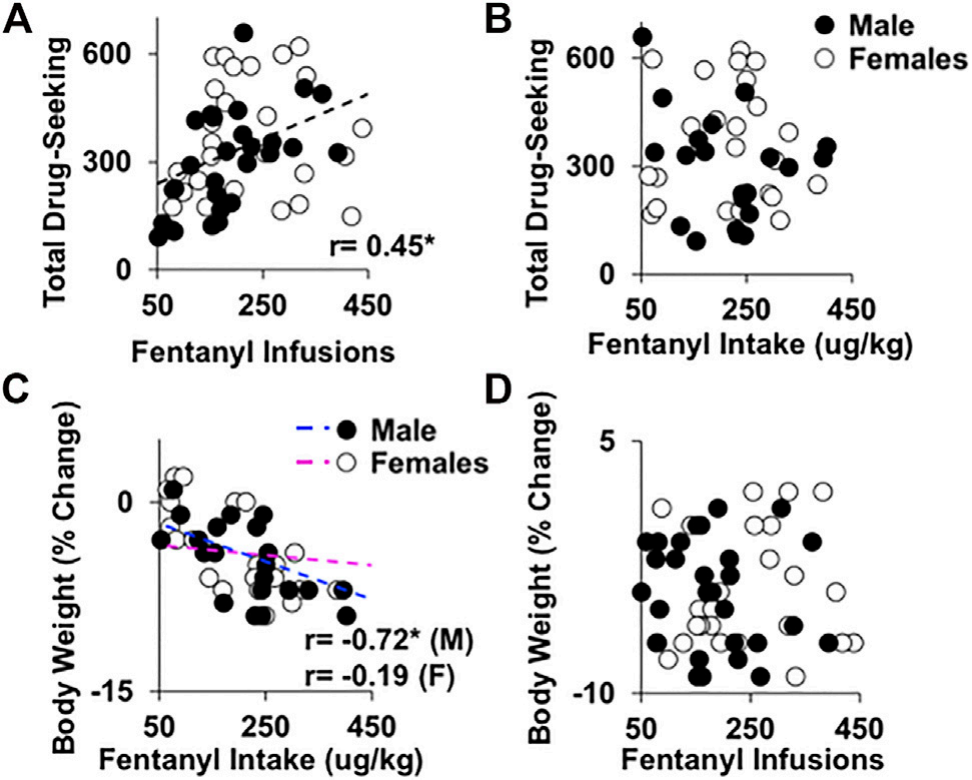
Associations between frequency of fentanyl use and relapse vulnerability and fentanyl intake and the development of physical dependence. Frequency of fentanyl use **(A)**, or average number of fentanyl infusions, but not amount of fentanyl use **(B)**, or average fentanyl intake, was positively correlated with relapse vulnerability during extinction and reinstatement testing (total responses) in both males and females (*n* = 29 males, *n* = 29 females). However, amount of fentanyl use **(C)**, but not frequency of fentanyl use **(D)**, was significantly correlated with development of physical dependence, or percent decrease in body weight during early withdrawal in males, but not in females (*n* = 29 males, *n* = 29 females). r, Pearson correlation, (*) significant association.

As predicted, frequency of fentanyl use, as defined by the average number of fentanyl infusions obtained during the extended-access period, was predictive of later relapse vulnerability during extinction and reinstatement testing (total responding; Figure 6A; r = 0.45, *p* < 0.001); this relationship was also similar between males and females (nonsignificant interaction of sex, *p* > 0.05). However, to our surprise, fentanyl intake (averaged across the extended-access period) was not significantly associated with relapse vulnerability in males or females (Figure 6B; *p*’s > 0.05). Although, as expected, the amount of fentanyl use was predictive of the development of physical dependence (Figure 6C), as defined by percent decrease in body weight during early withdrawal; however, this correlation was significant in males (r = −0.72, *p* < 0.001), but not in females (interaction of sex, F_1, 54_ = 5.5, *p* < 0.05). Importantly, this effect was specific to fentanyl intake, and not frequency of fentanyl use, given that the relationship between infusions and percent change in body weight during early withdrawal was nonsignificant for both males and females (Figure 6D; *p*’s > 0.05). Thus, frequency of opioid use, but not opioid intake, was predictive of relapse vulnerability in both males and females; whereas, opioid intake, but not frequency of use, was predictive of physical dependence in males, but not in females.

## Discussion

The goals of this study were to determine the fentanyl dose conditions that maximize the expression of an addiction-like phenotype in both males and females and to expand our model to include an additional key feature of OUD in humans, physical dependence. Surprisingly, despite markedly higher intake in groups given access to higher versus lower doses of fentanyl, each of the groups responded at similar levels during relapse testing (extinction and cue-induced reinstatement). We did observe a positive association between relapse vulnerability and number of infusions, but not fentanyl intake, indicating that frequency of use, but not total intake, impacts vulnerability to relapse. However, total intake was associated with the development of physical dependence given that weight loss was apparent following discontinuation of fentanyl self-administration at the three highest doses, but not following discontinuation of self-administration at the lowest dose. While this effect was similar between both males and females, the association between intake and weight loss was significant in males, but not females. Other notable sex differences were that both frequency of fentanyl use and intake were greater in females than in males, with particularly robust differences at lower doses, and that the time course for recovery of body weight loss during withdrawal was prolonged in females versus in males. As with our previous findings, females also had exhibited higher relapse vulnerability than males, particularly when they were tested during the estrus phase of their estrous cycle. Together, these findings indicate that sex is an important risk factor for pattern and levels of fentanyl intake, physical dependence, and relapse vulnerability, and while fentanyl intake predicts physical dependence, frequency of use predicts relapse vulnerability.

Contrary to our prediction, the dose of fentanyl self-administered during extended access did not impact subsequent vulnerability to relapse. This is surprising considering that fentanyl intake prior to withdrawal and relapse testing was markedly higher in the groups given access to higher versus lower doses of fentanyl. Our prediction that vulnerability to relapse would be enhanced in groups with higher versus lower intake of fentanyl (i.e., groups given access to higher versus lower doses of fentanyl) was based on reports in humans showing that the risk of relapse following treatment for OUD is higher in individuals who report higher levels of opioid use prior to treatment compared with those reporting lower levels (Gossop et al., 2002; Smyth et al., 2010; Grau-Lopez et al., 2021). While our findings appear to be in contrast to these results, it is important to note that these same studies also identified frequency of opioid use as a risk factor for relapse following treatment. It is also important to note that it is difficult in humans to determine levels of opioid use, since the dose is often unknown. For example, in one study (Smyth et al., 2010), the amount of heroin use prior to treatment was estimated based on the amount of the substance relative to a quarter. In this preclinical study, where levels and frequency of use were precisely measured, we found that frequency of use, but not levels of use, was predictive of vulnerability to relapse. Specifically, like findings in humans, we observed significant associations between frequency of use and relapse responses; rats in the lower fentanyl dose groups also obtained more infusions than rats in the higher dose groups and the highest relapse responses were observed in the groups given access to the lowest dose of fentanyl, not the highest dose (although this difference was not significant). Our interpretation of the association is also consistent with other studies with fentanyl and other addictive drugs showing that patterns of intake, but not amount of intake, are predictive of an enhanced vulnerability to relapse (Belin et al., 2009; Martin-Garcia et al., 2014; Allain and Samaha, 2019) as well as the development of an enhanced motivation for the drug (Zimmer et al., 2012; Allain et al., 2018; Fragale et al., 2021; Martin et al., 2021). The translational implication is that additional antirelapse interventions should be targeted toward individuals reporting high frequencies of opioid use and questions such as “how often” rather than “how much” may be more beneficial when trying to identify high-risk patients with OUD.

Intake was predictive of the development of physical dependence to fentanyl, and while the correlation between intake and weight loss was only significant in males, it is notable that in both sexes, physical dependence only developed following fentanyl self-administration at the three highest doses and neither males nor females showed weight loss during withdrawal from the 0.25-μg/kg dose. The significant correlation between intake and weight loss in males was expected based on multiple previous studies showing that rodents with greater opioid intake have more signs of physical dependence compared with rodents with less opioid intake (Vendruscolo et al., 2018; Towers et al., 2019; Moussawi et al., 2020). Clinical studies also report greater physical dependence in individuals reporting higher levels of opioid use (O’Malley and O’Malley, 2020; Rodríguez-Espinosa et al., 2021). However, it is somewhat surprising to observe it here considering that the weight loss observed during early withdrawal in males was similar between each of the three highest dose groups and fentanyl intake increased significantly with increases in the dose of fentanyl self-administered during extended access. Although, there was considerable variability in intake within dose groups, which likely accounts for this association in males. Interestingly, despite intake being greater in females compared with males, weight loss during early withdrawal was the same in females and males, which is similar to findings in mice where females had greater heroin intake over extended-access self-administration, but displayed a similar number of naloxone-precipitated withdrawal signs (Towers et al., 2019). This, along with weight loss not being significantly associated with physical dependence in females, is curious and suggests that physical dependence develops in females once a certain threshold of intake is achieved, but that, unlike effects in males, further increases in intake do not further enhance physical dependence in females.

Our findings showing that physical dependence developed in the three highest dose groups, but not the lowest dose group, yet each of the groups, including the lowest dose group, responded at similar levels during relapse testing suggest that the development of physical dependence is not necessary for the development of other addiction-like behaviors. This conclusion is supported by findings in humans showing that a substantial subgroup of people with OUD have relatively low levels of physical dependence (Kanof et al., 1991). Interestingly, a recent preclinical study (Townsend et al., 2021) also showed that despite similarities between men and women for both escalation of fentanyl intake and physical dependence, only the men developed an enhanced preference for fentanyl over a nondrug alternative reinforcer (Ensure). This is important because this shift is believed to represent another key feature of OUD in humans, an enhanced preference for a drug to the exclusion of other reinforcing stimuli and activities and further supports the notion that features of the addiction-like phenotype develop independently of one another. It also suggests that sex differences in vulnerability to OUD vary between different features of the disease (relapse, preference for the drug over other rewards, compulsive use, and motivation for the drug). This idea is also supported by our current findings showing that females were more vulnerable than males during relapse testing but did not differ from males for the expression of physical dependence during early withdrawal. It should be noted, however, that the conditions necessary for inducing addiction-like features with fentanyl have yet to be fully established. For example, we and others have shown that extended-access fentanyl self-administration induces an enhanced vulnerability to relapse when assessed following protracted abstinence; we also showed that this phenotype can be blocked by buprenorphine treatment during withdrawal. However, it is not clear if these effects differ from those observed following short-access self-administration, and the time–course for such changes during withdrawal have yet to be fully explored. A few studies have explored effects with other addiction-like features, although the Townsend study did show that the preference for the drug over a nondrug reinforcer was attenuated following treatment with methadone in men, but not in women, which further indicates that their model induced this feature of an addiction-like phenotype in men, but not in women. Further research is necessary to explore the fentanyl self-administration and withdrawal conditions necessary to induce addiction-like features in men and women.

Our findings also confirmed biological sex as an important vulnerability factor across the disease process. Specifically, during the extended-access phase, females self-administered more infusions and had higher levels of fentanyl intake than males, and as expected (Towers et al., 2019), these differences were most apparent under low dose conditions. These findings are consistent with our previous study with fentanyl self-administration under these extended-access conditions (Bakhti-Suroosh et al., 2021) and previous work with heroin showing robust sex differences in intake during extended-access self-administration under lower dose conditions (30 and 60 μg/kg/infusion; Towers et al., 2019), but not higher dose conditions (250 μg/kg/infusion; Zhang et al., 2015). These findings also provide insight as to why a few studies using higher doses of opioids have reported no sex differences in levels of opioid intake under extended-access conditions with heroin (100 μg/kg/infusion; Venniro et al., 2017; Venniro et al., 2019) and fentanyl (3.2 μg/kg/infusion, Townsend et al., 2021; 2.5 μg/kg/infusion, Hammerslag et al., 2021, Reiner et al., 2020). One exception was for binge intake, where females had higher intake than males regardless of dose. This is interesting because it suggests that even under high-dose conditions where intake is similar between males and females (Venniro et al., 2017; Venniro et al., 2019; Reiner et al., 2020; Hammerslag et al., 2021; Townsend et al., 2021), there is likely a sex difference in the pattern of self-administration. Indeed, numerous studies have shown that there are sex differences in patterns of extended-access drug self-administration under both high and low dose conditions (e.g., Lynch and Taylor, 2004; Towers et al., 2019) with female rodents self-administering more heroin during the first hour of a long, continuous access session (fixed-ratio 1, 6-h session; Towers et al., 2019), having a longer initial period of “binge” cocaine before taking a break and showing a more diurnal dysregulation pattern of cocaine use during 24-h/day sessions (Lynch and Taylor, 2004), and having greater binge-like alcohol drinking using a two-bottle, limited-access “drinking-in-the-dark” procedure (Sneddon et al., 2019) compared with males.

Females also showed an enhanced sensitivity to the anorectic effect of the low dose of fentanyl during extended-access self-administration. Most preclinical self-administration studies use 6- or 12-h extended-access sessions, which result in daily cycles of intake and withdrawal that are long enough to induce weight changes due to physical dependence (Townsend et al., 2021). This is the first study, to our knowledge, that has monitored changes in body weight in males and females over a period of 24-h/day opioid access followed by prolonged withdrawal. Under the lowest dose condition, females gained less percent body weight than males during both the first and last 5 days of extended-access self-administration. Although this effect may be driven by females having greater fentanyl intake than males under the lowest dose condition, weight gain in females was not further impacted with increases in fentanyl dose/intake like it was in males. In fact, males tended to have a smaller percent of body weight gained in the high dose conditions compared with females even though fentanyl intake was similar under these self-administration conditions. These anorectic effects of fentanyl are consistent with the side effects reported with the use of fentanyl patches in cancer patients (Wiffen et al., 2014), and while sex/gender differences have not been examined in humans, based on our findings, we would expect that this side effect might be more apparent in women at low doses and more apparent in men at high doses. Further research is necessary to examine these possibilities as dose-dependent side effects of opioid analgesics may have important sex differences that could impact patient care.

Although fentanyl intake was not correlated with weight loss during early withdrawal in females, there was an overall effect of dose with females self-administering the three highest doses of fentanyl, but not the lowest dose, losing a significant amount of their body weight during early withdrawal, similar to the findings in males. This finding, in addition to previous findings showing that weight loss occurs during early withdrawal (∼12 h) in females, similar to males (Townsend et al., 2021), and that the recovery of this weight loss follows a similar pattern as other somatic signs of opioid withdrawal in females (Bobzean et al., 2019), provides support for the use of weight loss as a measure of physical dependence in females. Interestingly, despite similar weight loss in males and females during early withdrawal from the three highest doses of fentanyl, females took longer to regain their body weight and continued to gain less body weight than males even during late withdrawal. These findings are consistent with another study that showed weight loss and somatic withdrawal symptoms, including stomach writhing symptoms, persisted longer in women compared with men following discontinuation of morphine administration (Bobzean et al., 2019) and findings in humans indicating that women experience a more severe withdrawal syndrome than men (Back et al., 2011b; Huhn et al., 2019; Dunn et al., 2020). These findings indicate that the physiological effects of opioid withdrawal may be prolonged in females compared with males. However, it is important to note that there are sex differences in weight gain under normal conditions, and these differences may contribute to the effects observed here during withdrawal. Future research that includes additional measures of physical dependence/opioid withdrawal is necessary to examine this possibility especially considering that the current withdrawal scales were developed using male animals. To our knowledge a detailed withdrawal syndrome following extended-access opioid self-administration remains unknown for females.

Finally, females showed an enhanced vulnerability during relapse testing compared with males, and these effects were particularly robust during extinction testing and when females were tested during estrus versus non-estrus phases of their estrous cycle. These results were expected based on our previous findings with fentanyl (Bakhti-Suroosh et al., 2021). They are also consistent with results with other addictive drugs showing greater cue-induced reinstatement responding in estrus females compared with non-estrus females and males during late and protracted withdrawal (days 15, 30, and 48; Nicolas et al., 2019: Corbett et al., 2021); although here, there was only a trend for estrus females having higher reinstatement versus males, extinction responding was significantly higher in estrus females compared with non-estrus females and males. This is important considering that a number of recent studies have reported that sex differences are not relevant for cue-induced relapse/reinstatement with opioids (Cooper et al., 2007; Venniro et al., 2017; Venniro et al., 2019; Fredriksson et al., 2020; Reiner et al., 2020), yet differences were likely due to inclusion of a greater percentage of non-estrus versus estrus females. Importantly, our preclinical findings are consentient with clinical findings showing that women have greater opioid (Yu et al., 2007; Kennedy et al., 2013; Moran et al., 2018), alcohol (Willner et al., 1998; Seo et al., 2011), and cocaine (Robbins et al., 1999) craving in the presence of drug-associated cues compared with men (but see Avants et al., 1995; Volkow et al., 2011). These sex/gender differences in humans have also attributed, at least in part, to hormonal changes over the menstrual cycle given that the positive subjective effects of cocaine tend to increase with higher levels of estradiol during the follicular phase and to be reduced when progesterone is higher during the luteal phase (Fox et al., 2008). Although similar effects have not been observed in women with OUD, the menstrual cycle is disrupted by opioid use making it difficult to determine the impact of gonadal hormones on drug craving and use (Santen et al., 1975). Our findings indicate that gonadal hormones likely impact relapse vulnerability with opioids given that extinction and reinstatement responding was higher in females tested during estrus, when the ratio of estradiol to progesterone is relatively high, versus non-estrus females. Future studies investigating relapse vulnerability in females, and particularly those that examine effects within one test session, should consider hormonal status prior to making conclusions regarding sex differences (or lack thereof) as the proportion of females tested during estrus versus non-estrus phases (or luteal versus follicular phases) could drastically change the results and conclusions.

In summary, our findings indicate that patterns of use, rather than absolute levels of use, impact vulnerability to relapse. Females also showed an enhanced vulnerability to relapse compared with males, particularly when they were tested during estrus. The translational implications of our findings are that additional antirelapse intervention may be necessary for women and individuals reporting high frequencies of opioid use. These findings also have implications for studies on sex/gender differences in substance-use disorder as they further support the idea that sex differences are most apparent under low-dose conditions that induce intersubject variability. They also indicate that sex/gender differences may be apparent for patterns of intake even in the absence of a sex difference for overall levels of use; this is important considering that the pattern of use, but not intake, was predictive of relapse vulnerability. Finally, they indicate that a lack of a sex/gender difference for relapse vulnerability during extinction/reinstatement testing may be due to the distribution of females tested at different menstrual/estrus cycle. Thus, phase of menstrual/estrous cycle should be considered in studies of sex differences in relapse.

## Data Availability

The raw data supporting the conclusions of this article will be made available by the authors, without undue reservation.

## References

[B1] Adelson M. Linzy S. Peles E. (2018). Characteristics and Outcome of Male and Female Methadone Maintenance Patients: MMT in Tel Aviv and Las Vegas. Subst. Use Misuse. 53, 230–238. 10.1080/10826084.2017.1298619 28574738

[B2] Allain F. Bouayad-Gervais K. Samaha A. N. (2018). High and Escalating Levels of Cocaine Intake Are Dissociable from Subsequent Incentive Motivation for the Drug in Rats. Psychopharmacology (Berl). 235, 317–328. 10.1007/s00213-017-4773-8 29085961

[B3] Allain F. Samaha A. N. (2019). Revisiting Long-Access versus Short-Access Cocaine Self-Administration in Rats: Intermittent Intake Promotes Addiction Symptoms Independent of Session Length. Addict. Biol. 24, 641–651. 10.1111/adb.12629 29920865

[B4] American Psychiatric Association (2013). “Substance-related and Addictive Disorders,” in Diagnostic and Statistical Manual of Mental Disorders. 5th ed. (Arlington, VA: American Psychiatric Association).

[B5] Anglin M. D. Hser Y. I. McGlothlin W. H. (1987). Sex Differences in Addict Careers. 2. Becoming Addicted. Am. J. Drug Alcohol. Abuse. 13, 59–71. 10.3109/00952998709001500 3687885

[B6] Avants S. K. Margolin A. Kosten T. R. Cooney N. L. (1995). Differences Between Responders and Nonresponders to Cocaine Cues in the Laboratory. Addict. Behav. 20, 215–224. 10.1016/0306-4603(94)00066-2 7484315

[B7] Back S. E. Payne R. L. Wahlquist A. H. Carter R. E. Stroud Z. Haynes L. (2011a). Comparative Profiles of Men and Women with Opioid Dependence: Results from a National Multisite Effectiveness Trial. Am. J. Drug Alcohol. Abuse. 37, 313–323. 10.3109/00952990.2011.596982 21854273 PMC3164783

[B8] Back S. E. Lawson K. M. Singleton L. M. Brady K. T. (2011b). Characteristics and Correlates of Men and Women with Prescription Opioid Dependence. Addict. Behav. 36, 829–834. 10.1016/j.addbeh.2011.03.013 21514061 PMC3164361

[B9] Bakhti-Suroosh A. Towers E. B. Lynch W. J. (2021). A Buprenorphine-Validated Rat Model of Opioid Use Disorder Optimized to Study Sex Differences in Vulnerability to Relapse. Psychopharmacology (Berl). 238, 1029–1046. 10.1007/s00213-020-05750-2 33404740 PMC7786148

[B78] Beiter R. M. Peterson A. B. Abel J. Lynch W. J. (2016). Exercise During Early, but not Late Abstinence, Attenuates Subsequent Relapse Vulnerability in a Rat Model. Transl Psychiatry 6, e792–e792. 10.1038/tp.2016.58 27115123 PMC4872415

[B10] Belin D. Balado E. Piazza P. V. Deroche-Gamonet V. (2009). Pattern of Intake and Drug Craving Predict the Development of Cocaine Addiction-like Behavior in Rats. Biol. Psychiatry. 65, 863–868. 10.1016/j.biopsych.2008.05.031 18639867

[B11] Bobzean S. A. M. Kokane S. S. Butler B. D. Perrotti L. I. (2019). Sex Differences in the Expression of Morphine Withdrawal Symptoms and Associated Activity in the Tail of the Ventral Tegmental Area. Neurosci. Lett. 705, 124–130. 10.1016/j.neulet.2019.04.057 31042569 PMC6662583

[B12] Carroll M. E. Lynch W. J. Roth M. E. Morgan A. D. Cosgrove K. P. (2004). Sex and Estrogen Influence Drug Abuse. Trends Pharmacol. Sci. 25, 273–279. 10.1016/j.tips.2004.03.011 15120494

[B13] Centers for Disease Control and Prevention (2021). Drug Overdose Deaths in the U.S Top 100,000 Annually. National Center for Health Statistics, Office of Communication

[B14] Cicero T. J. Meyer E. R. (1973). Morphine Pellet Implantation in Rats: Quantitative Assessment of Tolerance and Dependence. J. Pharmacol. Exp. Ther. 184, 404–408. 4734581

[B15] Cooper A. Barnea-Ygael N. Levy D. Shaham Y. Zangen A. (2007). A Conflict Rat Model of Cue-Induced Relapse to Cocaine Seeking. Psychopharmacology (Berl). 194, 117–125. 10.1007/s00213-007-0827-7 17558499 PMC3733223

[B16] Corbett C. M. Dunn E. Loweth J. A. (2021). Effects of Sex and Estrous Cycle on the Time Course of Incubation of Cue-Induced Craving Following Extended-Access Cocaine Self-Administration. eNeuro. 8, ENEURO.0054-21.2021. 10.1523/ENEURO.0054-21.2021 PMC8362687 34290059

[B17] Dao A. N. Beacher N. J. Mayr V. Montemarano A. Hammer S. West M. O. (2021). Chronic Fentanyl Self-Administration Generates a Shift toward Negative Affect in Rats during Drug Use. Brain Sci. 11, 1064. 10.3390/brainsci11081064 34439683 PMC8394963

[B18] Dunn K. E. Weerts E. M. Huhn A. S. Schroeder J. R. Tompkins D. A. Bigelow G. E. (2020). Preliminary Evidence of Different and Clinically Meaningful Opioid Withdrawal Phenotypes. Addict. Biol. 25, e12680. 10.1111/adb.12680 30295400 PMC6546557

[B19] Fox H. C. Hong K. A. Paliwal P. Morgan P. T. Sinha R. (2008). Altered Levels of Sex and Stress Steroid Hormones Assessed Daily over a 28-day Cycle in Early Abstinent Cocaine-dependent Females. Psychopharmacology (Berl). 195, 527–536. 10.1007/s00213-007-0936-3 17891383 PMC2746368

[B20] Fragale J. E. James M. H. Aston-Jones G. (2021). Intermittent Self-Administration of Fentanyl Induces a Multifaceted Addiction State Associated with Persistent Changes in the Orexin System. Addict. Biol. 26, e12946. 10.1111/adb.12946 32798290 PMC7882007

[B21] Fredriksson I. Applebey S. V. Minier-Toribio A. Shekara A. Bossert J. M. Shaham Y. (2020). Effect of the Dopamine Stabilizer (-)-OSU6162 on Potentiated Incubation of Opioid Craving after Electric Barrier-Induced Voluntary Abstinence. Neuropsychopharmacology. 45, 770–779. 10.1038/s41386-020-0602-6 31905372 PMC7075949

[B22] Gellert V. F. Holtzman S. G. (1978). Development and Maintenance of Morphine Tolerance and Dependence in the Rat by Scheduled Access to Morphine Drinking Solutions. J. Pharmacol. Exp. Ther. 205, 536–546. 566320

[B23] Gossop M. Stewart D. Browne N. Marsden J. (2002). Factors Associated with Abstinence, Lapse or Relapse to Heroin Use after Residential Treatment: Protective Effect of Coping Responses. Addiction. 97, 1259–1267. 10.1046/j.1360-0443.2002.00227.x 12359030

[B24] Grau-López L. Roncero C. Daigre C. Gonzalvo B. Bachiller D. Rodriguez-Cintas L. (2012). Risk Factors for Relapse in Drug-dependent Patients after Hospital Detoxification. Adicciones. 24, 115–122. 22648314

[B25] Hammerslag L. R. Denehy E. D. Carper B. Nolen T. L. Prendergast M. A. Bardo M. T. (2021). Effects of the Glucocorticoid Receptor Antagonist PT150 on Stress-Induced Fentanyl Seeking in Male and Female Rats. Psychopharmacology (Berl). 238, 2439–2447. 10.1007/s00213-021-05865-0 34008048 PMC10323366

[B26] Hernandez-Avila C. A. Rounsaville B. J. Kranzler H. R. (2004). Opioid-, Cannabis- and Alcohol-dependent Women Show More Rapid Progression to Substance Abuse Treatment. Drug Alcohol Depend. 74, 265–272. 10.1016/j.drugalcdep.2004.02.001 15194204

[B27] Houshyar H. Manalo S. Dallman M. F. (2004). Time-Dependent Alterations in mRNA Expression of Brain Neuropeptides Regulating Energy Balance and Hypothalamo-Pituitary-Adrenal Activity after Withdrawal from Intermittent Morphine Treatment. J. Neurosci. 24, 9414–9424. 10.1523/JNEUROSCI.1641-04.2004 15496677 PMC6730111

[B28] Hser Y. I. Anglin M. D. McGlothlin W. (1987). Sex Differences in Addict Careers. 1. Initiation of Use. Am. J. Drug Alcohol. Abuse. 13, 33–57. 10.3109/00952998709001499 3318399

[B29] Huhn A. S. Dunn K. E. (2020). Challenges for Women Entering Treatment for Opioid Use Disorder. Curr. Psychiatry Rep. 22, 76. 10.1007/s11920-020-01201-z 33128093

[B30] Huhn A. S. Tompkins D. A. Campbell C. M. Dunn K. E. (2019). Individuals with Chronic Pain Who Misuse Prescription Opioids Report Sex-Based Differences in Pain and Opioid Withdrawal. Pain Med. 20, 1942–1947. 10.1093/pm/pny295 30690594 PMC6784741

[B31] Kanof P. D. Aronson M. J. Ness R. Cochrane K. J. Horvath T. B. Handelsman L. (1991). Levels of Opioid Physical Dependence in Heroin Addicts. Drug Alcohol Depend. 27, 253–262. 10.1016/0376-8716(91)90008-m 1884668

[B32] Kennedy A. P. Epstein D. H. Phillips K. A. Preston K. L. (2013). Sex Differences in Cocaine/heroin Users: Drug-Use Triggers and Craving in Daily Life. Drug Alcohol Depend. 132, 29–37. 10.1016/j.drugalcdep.2012.12.025 23357742 PMC3664120

[B33] Kerstetter K. A. Aguilar V. R. Parrish A. B. Kippin T. E. (2008). Protracted Time-dependent Increases in Cocaine-Seeking Behavior during Cocaine Withdrawal in Female Relative to Male Rats. Psychopharmacology (Berl). 198, 63–75. 10.1007/s00213-008-1089-8 18265959

[B34] Lacy R. T. Austin B. P. Strickland J. C. (2020). The Influence of Sex and Estrous Cyclicity on Cocaine and Remifentanil Demand in Rats. Addict. Biol. 25, e12716. 10.1111/adb.12716 30779409 PMC6916383

[B35] Lynch W. J. (2008). Acquisition and Maintenance of Cocaine Self-Administration in Adolescent Rats: Effects of Sex and Gonadal Hormones. Psychopharmacology (Berl). 197, 237–246. 10.1007/s00213-007-1028-0 18066534

[B36] Lynch W. J. Carroll M. E. (2001). Regulation of Drug Intake. Exp. Clin. Psychopharmacol. 9, 131–143. 10.1037//1064-1297.9.2.131 11518086

[B79] Lynch W. J. Taylor J. R. (2004). Sex Differences in the Behavioral Effects of 24-h/day Access to Cocaine Under a Discrete Rrial Procedure. Neuropsychopharmacology 29, 943–951. 10.1038/sj.npp.1300389 14872204

[B37] Lynch W. J. Tan L. Narmeen S. Beiter R. Brunzell D. H. (2019). Exercise or Saccharin during Abstinence Block Estrus-Induced Increases in Nicotine-Seeking. Physiol. Behav. 203, 33–41. 10.1016/j.physbeh.2017.10.026 29080668 PMC5927845

[B38] Maldonado R. Fournié-Zaluski M. C. Roques B. P. (1992). Attenuation of the Morphine Withdrawal Syndrome by Inhibition of Catabolism of Endogenous Enkephalins in the Periaqueductal gray Matter. Naunyn Schmiedebergs Arch. Pharmacol. 345, 466–472. 10.1007/BF00176626 1620246

[B39] Malone S. G. Keller P. S. Hammerslag L. R. Bardo M. T. (2021). Escalation and Reinstatement of Fentanyl Self-Administration in Male and Female Rats. Psychopharmacology (Berl). 238, 2261–2273. 10.1007/s00213-021-05850-7 33895852 PMC10332850

[B40] Mantsch J. R. Yuferov V. Mathieu-Kia A. M. Ho A. Kreek M. J. (2004). Effects of Extended Access to High versus Low Cocaine Doses on Self-Administration, Cocaine-Induced Reinstatement and Brain mRNA Levels in Rats. Psychopharmacology (Berl). 175, 26–36. 10.1007/s00213-004-1778-x 15042275

[B41] Marsh J. C. Park K. Lin Y. A. Bersamira C. (2018). Gender Differences in Trends for Heroin Use and Nonmedical Prescription Opioid Use, 2007-2014. J. Subst. Abuse Treat. 87, 79–85. 10.1016/j.jsat.2018.01.001 29433788 PMC9084392

[B42] Martin D. A. Gyawali U. Calu D. J. (2021). Effects of 5-HT2A Receptor Stimulation on Economic Demand for Fentanyl after Intermittent and Continuous Access Self-Administration in Male Rats. Addict. Biol. 26, e12926. 10.1111/adb.12926 32458577 PMC7688480

[B43] Martín-García E. Courtin J. Renault P. Fiancette J. F. Wurtz H. Simonnet A. (2014). Frequency of Cocaine Self-Administration Influences Drug Seeking in the Rat: Optogenetic Evidence for a Role of the Prelimbic Cortex. Neuropsychopharmacology. 39, 2317–2330. 10.1038/npp.2014.66 24633559 PMC4138740

[B44] Mazure C. M. Fiellin D. A. (2018). Women and Opioids: Something Different Is Happening Here. Lancet. 392, 9–11. 10.1016/S0140-6736(18)31203-0 30047402

[B45] Moran L. M. Kowalczyk W. J. Phillips K. A. Vahabzadeh M. Lin J. L. Mezghanni M. (2018). Sex Differences in Daily Life Stress and Craving in Opioid-dependent Patients. Am. J. Drug Alcohol. Abuse. 44, 512–523. 10.1080/00952990.2018.1454934 29641291 PMC6159214

[B46] Morgan A. D. Campbell U. C. Fons R. D. Carroll M. E. (2002). Effects of Agmatine on the Escalation of Intravenous Cocaine and Fentanyl Self-Administration in Rats. Pharmacol. Biochem. Behav. 72, 873–880. 10.1016/s0091-3057(02)00774-8 12062577

[B47] Moussawi K. Ortiz M. M. Gantz S. C. Tunstall B. J. Marchette R. C. N. Bonci A. (2020). Fentanyl Vapor Self-Administration Model in Mice to Study Opioid Addiction. Sci. Adv. 6, eabc0413. 10.1126/sciadv.abc0413 32821843 PMC7406365

[B48] National Institute on Drug Abuse (2021). Notice of Special Interest (NOSI): Administrative Supplements for Research on Fentanyl and Derivatives. NIH Guide for Grants and Contracts

[B49] National Survey on Drug Use and Health (2018). Concatenated Public Use File (2002 to 2018). Available at: https://pdas.samhsa.gov/#/ (Accessed February, , 2020).

[B50] Navarro-Zaragoza J. Núñez C. Laorden M. L. Milanés M. V. (2010). Effects of Corticotropin-Releasing Factor Receptor-1 Antagonists on the Brain Stress System Responses to Morphine Withdrawal. Mol. Pharmacol. 77, 864–873. 10.1124/mol.109.062463 20159948

[B51] Nickel B. Aledter A. (1987). Comparative Physical Dependence Studies in Rats with Flupirtine and Opiate Receptor Stimulating Analgesics. Postgrad. Med. J. 63 (Suppl. 3), 41–43. 2833735

[B52] Nicolas C. Russell T. I. Pierce A. F. Maldera S. Holley A. You Z. B. (2019). Incubation of Cocaine Craving after Intermittent-Access Self-Administration: Sex Differences and Estrous Cycle. Biol. Psychiatry. 85, 915–924. 10.1016/j.biopsych.2019.01.015 30846301 PMC6534474

[B53] O’Malley G. F. O’Malley R. (2020). Opioid Toxicity and Withdrawal. Merck Manual.

[B54] Peterson A. B. Hivick D. P. Lynch W. J. (2014). Dose-Dependent Effectiveness of Wheel Running to Attenuate Cocaine-Seeking: Impact of Sex and Estrous Cycle in Rats. Psychopharmacology (Berl). 231, 2661–2670. 10.1007/s00213-014-3437-1 24464528

[B55] Pintér-Kübler B. Ferenczi S. Núnez C. Zelei E. Polyák A. Milanés M. V. (2013). Differential Changes in Expression of Stress- and Metabolic-Related Neuropeptides in the Rat Hypothalamus during Morphine Dependence and Withdrawal. PLoS One. 8, e67027. 10.1371/journal.pone.0067027 23805290 PMC3689674

[B56] Reiner D. J. Lofaro O. M. Applebey S. V. Korah H. Venniro M. Cifani C. (2020). Role of Projections between Piriform Cortex and Orbitofrontal Cortex in Relapse to Fentanyl Seeking after Palatable Food Choice-Induced Voluntary Abstinence. J. Neurosci. 40, 2485–2497. 10.1523/JNEUROSCI.2693-19.2020 32051327 PMC7083529

[B57] Robbins S. J. Ehrman R. N. Childress A. R. O'Brien C. P. (1999). Comparing Levels of Cocaine Cue Reactivity in Male and Female Outpatients. Drug Alcohol Depend. 53, 223–230. 10.1016/s0376-8716(98)00135-5 10080048

[B58] Rodríguez-Espinosa S. Coloma-Carmona A. Pérez-Carbonell A. Román-Quiles J. F. Carballo J. L. (2021). Clinical and Psychological Factors Associated with Interdose Opioid Withdrawal in Chronic Pain Population. J. Subst. Abuse Treat. 129, 108386. 10.1016/j.jsat.2021.108386 34080554

[B77] Sanchez V. Moore C. F. Brunzell D. H. Lynch W. J. (2014). Sex Differences in the Effect of Wheel Running on Subsequent Nicotine-Seeking in a Rat Adolescent-Onset Self-Administration Model. Psychopharmacology 231, 1753–1762. 10.1007/s00213-013-3359-3 24271035 PMC3969388

[B59] Santen F. J. Sofsky J. Bilic N. Lippert R. (1975). Mechanism of Action of Narcotics in the Production of Menstrual Dysfunction in Women. Fertil. Steril. 26 (6), 538–548. 10.1016/s0015-0282(16)41173-8 236938

[B60] Seaman R. W. Jr. Collins G. T. (2021). Impact of Morphine Dependence and Withdrawal on the Reinforcing Effectiveness of Fentanyl, Cocaine, and Methamphetamine in Rats. Front. Pharmacol. 12, 691700. 10.3389/fphar.2021.691700 34093214 PMC8175987

[B61] Seo D. Jia Z. Lacadie C. M. Tsou K. A. Bergquist K. Sinha R. (2011). Sex Differences in Neural Responses to Stress and Alcohol Context Cues. Hum. Brain Mapp. 32 (11), 1998–2013. 10.1002/hbm.21165 21162046 PMC3236497

[B62] Smyth B. P. Barry J. Keenan E. Ducray K. (2010). Lapse and Relapse Following Inpatient Treatment of Opiate Dependence. Ir Med. J. 103, 176–179. 20669601

[B63] Sneddon E. A. White R. D. Radke A. K. (2019). Sex Differences in Binge-like and Aversion-Resistant Alcohol Drinking in C57BL/6J Mice. Alcohol. Clin. Exp. Res. 43 (2), 243–249. 10.1111/acer.13923 30431655

[B64] Torres O. V. Walker E. M. Beas B. S. O'Dell L. E. (2014). Female Rats Display Enhanced Rewarding Effects of Ethanol that Are Hormone Dependent. Alcohol. Clin. Exp. Res. 38, 108–115. 10.1111/acer.12213 23909760 PMC3842413

[B65] Towers E. B. Tunstall B. J. McCracken M. L. Vendruscolo L. F. Koob G. F. (2019). Male and Female Mice Develop Escalation of Heroin Intake and Dependence Following Extended Access. Neuropharmacology. 151, 189–194. 10.1016/j.neuropharm.2019.03.019 30880124 PMC9345532

[B66] Townsend E. A. Kim R. K. Robinson H. L. Marsh S. A. Banks M. L. Hamilton P. J. (2021). Opioid Withdrawal Produces Sex-Specific Effects on fentanyl-vs.-food Choice and Mesolimbic Transcription. Biol. Psychiatry Glob. Open Sci. 1, 112–122. 10.1016/j.bpsgos.2021.04.009 34458885 PMC8389189

[B67] Vendruscolo J. C. M. Tunstall B. J. Carmack S. A. Schmeichel B. E. Lowery-Gionta E. G. Cole M. (2018). Compulsive-Like Sufentanil Vapor Self-Administration in Rats. Neuropsychopharmacology. 43, 801–809. 10.1038/npp.2017.172 28812595 PMC5809787

[B68] Venniro M. Russell T. I. Zhang M. Shaham Y. (2019). Operant Social Reward Decreases Incubation of Heroin Craving in Male and Female Rats. Biol. Psychiatry. 86, 848–856. 10.1016/j.biopsych.2019.05.018 31326085 PMC8383184

[B69] Venniro M. Zhang M. Shaham Y. Caprioli D. (2017). Incubation of Methamphetamine but Not Heroin Craving after Voluntary Abstinence in Male and Female Rats. Neuropsychopharmacology. 42, 1126–1135. 10.1038/npp.2016.287 28025975 PMC5506794

[B70] Volkow N. D. Tomasi D. Wang G. J. Fowler J. S. Telang F. Goldstein R. Z. (2011). Reduced Metabolism in Brain "Control Networks" Following Cocaine-Cues Exposure in Female Cocaine Abusers. PLoS One. 6, e16573. 10.1371/journal.pone.0016573 21373180 PMC3043072

[B71] Wade C. L. Vendruscolo L. F. Schlosburg J. E. Hernandez D. O. Koob G. F. (2015). Compulsive-like Responding for Opioid Analgesics in Rats with Extended Access. Neuropsychopharmacology. 40, 421–428. 10.1038/npp.2014.188 25060491 PMC4443956

[B72] Wiffen P. J. Derry S. Moore R. A. (2014). Impact of Morphine, Fentanyl, Oxycodone or Codeine on Patient Consciousness, Appetite and Thirst when Used to Treat Cancer Pain. Cochrane Database Syst. Rev. 2014 (5), CD011056. 10.1002/14651858.CD011056.pub2 PMC6483540 24874470

[B73] Willner P. Field M. Pitts K. Reeve G. (1998). Mood, Cue and Gender Influences on Motivation, Craving and Liking for Alcohol in Recreational Drinkers. Behav. Pharmacol. 9, 631–642. 10.1097/00008877-199811000-00018 9862088

[B74] Yu J. Zhang S. Epstein D. H. Fang Y. Shi J. Qin H. (2007). Gender and Stimulus Difference in Cue-Induced Responses in Abstinent Heroin Users. Pharmacol. Biochem. Behav. 86, 485–492. 10.1016/j.pbb.2007.01.008 17306353

[B75] Zhang Y. Picetti R. Butelman E. R. Ho A. Blendy J. A. Kreek M. J. (2015). Mouse Model of the OPRM1 (A118G) Polymorphism: Differential Heroin Self-Administration Behavior Compared with Wild-Type Mice. Neuropsychopharmacology. 40, 1091–1100. 10.1038/npp.2014.286 25336208 PMC4367451

[B76] Zimmer B. A. Oleson E. B. Roberts D. C. (2012). The Motivation to Self-Administer Is Increased after a History of Spiking Brain Levels of Cocaine. Neuropsychopharmacology. 37, 1901–1910. 10.1038/npp.2012.37 22453139 PMC3376322

